# Differential GR Expression and Translocation in the Hippocampus Mediates Susceptibility vs. Resilience to Chronic Social Defeat Stress

**DOI:** 10.3389/fnins.2017.00287

**Published:** 2017-05-23

**Authors:** Qiu-Qin Han, Liu Yang, Hui-Jie Huang, Ya-Lin Wang, Rui Yu, Jing Wang, Adam Pilot, Gen-Cheng Wu, Qiong Liu, Jin Yu

**Affiliations:** ^1^Department of Integrative Medicine and Neurobiology, State Key Laboratory of Medical Neurobiology, School of Basic Medical Sciences, Institutes of Brain Science, Brain Science Collaborative Innovation Center, Shanghai Medical College, Fudan UniversityShanghai, China; ^2^Department of Anatomy, Histology and Embryology, School of Basic Medical Sciences, Shanghai Medical College, Fudan UniversityShanghai, China; ^3^Key Laboratory of Medical Imaging Computing and Computer Assisted Intervention of ShanghaiShanghai, China

**Keywords:** social defeat stress, susceptibility, resilience, HPA axis, CRF, corticosterone, GR

## Abstract

While social stress exposure is a common risk factor for affective disorders, most individuals exposed to it can maintain normal physical and psychological functioning. However, factors that determine susceptibility vs. resilience to social stress remain unclear. Here, the resident-intruder model of social defeat was used as a social stressor in male C57BL/6J mice to investigate the difference between susceptibility and resilience. As depression is often characterized by hyperactivity of the hypothalamic-pituitary-adrenal (HPA) axis, we conducted the present study to further investigate the individual differences in the HPA axis response and glucocorticoid receptor (GR) protein expression and translocation between susceptible mice and resilient mice. We found that hypercortisolemia, induced by social defeat stress occurred in susceptible mice, but not in resilient mice. Moreover, susceptible mice exhibited significantly less GR protein expression and nuclear translocation in the hippocampus than resilient mice. Treatment with escitalopram could decrease the serum corticosterone (CORT), increase GR protein expression as well as nuclear translocation in the hippocampus and ultimately reverse social withdrawal behaviors in susceptible mice. These results indicate that the up-regulation of GR and the enhancement of GR nuclear translocation in the hippocampus play an important role in resilience to chronic social defeat stress.

## Introduction

Depression is a public health concern associated with high morbidity and mortality (Silva et al., 2014). The prevalence of depression morbidity is increasing (Compton et al., 2006), and by 2020, depression is estimated to be the second most common cause of disability. Historically, to induce depression in animals, various forms of chronic stress have been utilized. The most typical and popular models are chronic social defeat stress and chronic unpredictable stress (Nestler and Hyman, 2010). According to the standardized protocol for repeated social defeat stress in mice published in Nature Protocols, C57BL/6J mice are repeatedly subjected to bouts of social defeat (Golden et al., 2011). Chronic social stress results in serious effects (Fuchs and Flugge, 2002) such as a robust depression-like phenotype marked by passive defense postures, social-avoidance behaviors, anhedonia, anxiety (Kudryavtseva et al., 1991; Rygula et al., 2005), and cognitive dysfunction (Yu et al., 2011). Antidepressants can counteract these depressive-like symptoms (Berton et al., 2006; Rygula et al., 2006a,b). Clinically, depression is often characterized by hyperactivity of the hypothalamic-pituitary-adrenal (HPA) axis (Schuhmacher et al., 2013; Schatzberg, 2015). In animal research, numerous studies also reveal that the HPA axis shows an activated response when rats or mice experience social defeat stress (Pich et al., 1993; Keeney et al., 2006; Razzoli et al., 2009), which can be relieved by antidepressants (Becker et al., 2008). The activation of the HPA axis is controlled by glucocorticoid receptor (GR), acting as a ligand-activated transcription factor, which translocates from the cytosol to the nucleus through a negative feedback mechanism at different levels (McEwen et al., 1992). The expression and translocation of GR has been found to be disturbed in response to stress (Mizoguchi et al., 2003; Raone et al., 2007; Guidotti et al., 2013). These studies reveal that the disturbance of the HPA axis response and GR protein expression and translocation may relevantly correlate with some of the pathological abnormalities observed in depression.

Stress exposure can be differently perceived by individuals and it can have long-term sequelae depending on its level (Franklin et al., 2012). In most cases, human beings resist the development of neuropsychiatric disorders in the face of stress. This is commonly referred to as “resilience.” It is defined as a trait of individuals with the capacity to avoid negative biological, psychological and social consequences of irritable conditions (Russo et al., 2012). Furthermore, recent reports indicate that resilience represents an adaptive process shaping the active functioning of neural circuits to mediate successful coping with stress (Charney, 2004; Feder et al., 2009). In a word, resilient individuals can perceive irritable conditions as minor stress and develop adaptive responses (Del et al., 2011). Thus, there is a question: How do most people manage to maintain normal physiological and psychological function when exposed even to extraordinary levels of stress while others not? Characterizing biological factors associated with more successful coping responses in resilient individuals, surely means a lot to the susceptible ones. According to the standardized protocol for repeated social defeat stress which has been mentioned above, the defeated mice can be classified as susceptible ones that exhibit social avoidance and resilient ones that fail to develop such avoidance (Golden et al., 2011). The behavioral differences make the social defeat model useful in studying individual differences in the face of extreme stress.

Therefore, we conducted the present study to further investigate the individual differences in the HPA axis response and GR protein expression and translocation between susceptible mice and resilient mice. To assess the behavioral abnormalities, we carried out social interaction test; to monitor the HPA axis response, we detected the mRNA expression of the corticotropin-releasing factor (*Crf*) in the hypothalamus and serum corticosterone (CORT); to evaluate the regulation of HPA axis, the GR protein expression in the hippocampus and hypothalamus as well as the GR protein translocation in the hippocampus were studied. Besides, we also injected susceptible mice with escitalopram, an antidepressant, to observe if it could counteract behavioral abnormalities and the disturbance of the HPA axis response and GR protein expression and translocation in susceptible mice or not.

## Materials and methods

### Animals

For all experiments, male C57BL/6J mice (7–8 weeks, from Shanghai Laboratory Animal Center, Chinese Academy of Sciences, China) were fed *ad libitum*, allowed a 1 week habituation period before experimental manipulation, and housed at 23 ± 2°C on a 12 h light/dark cycle (lights on at 07:00). CD-1 retired breeders (male, 8–9 months, from Vital River Laboratories, Beijing, China) were used as the aggressors and were screened every 3 months to ensure their antagonistic interactions. This study was carried out in accordance with the National Institute of Health Guide for the Care and Use of Laboratory Animals. The protocol was approved by Animal Ethics Committee of Shanghai Medical College, Fudan University, Shanghai, China (20120302-107).

### Social defeat stress

**Figure 1:**
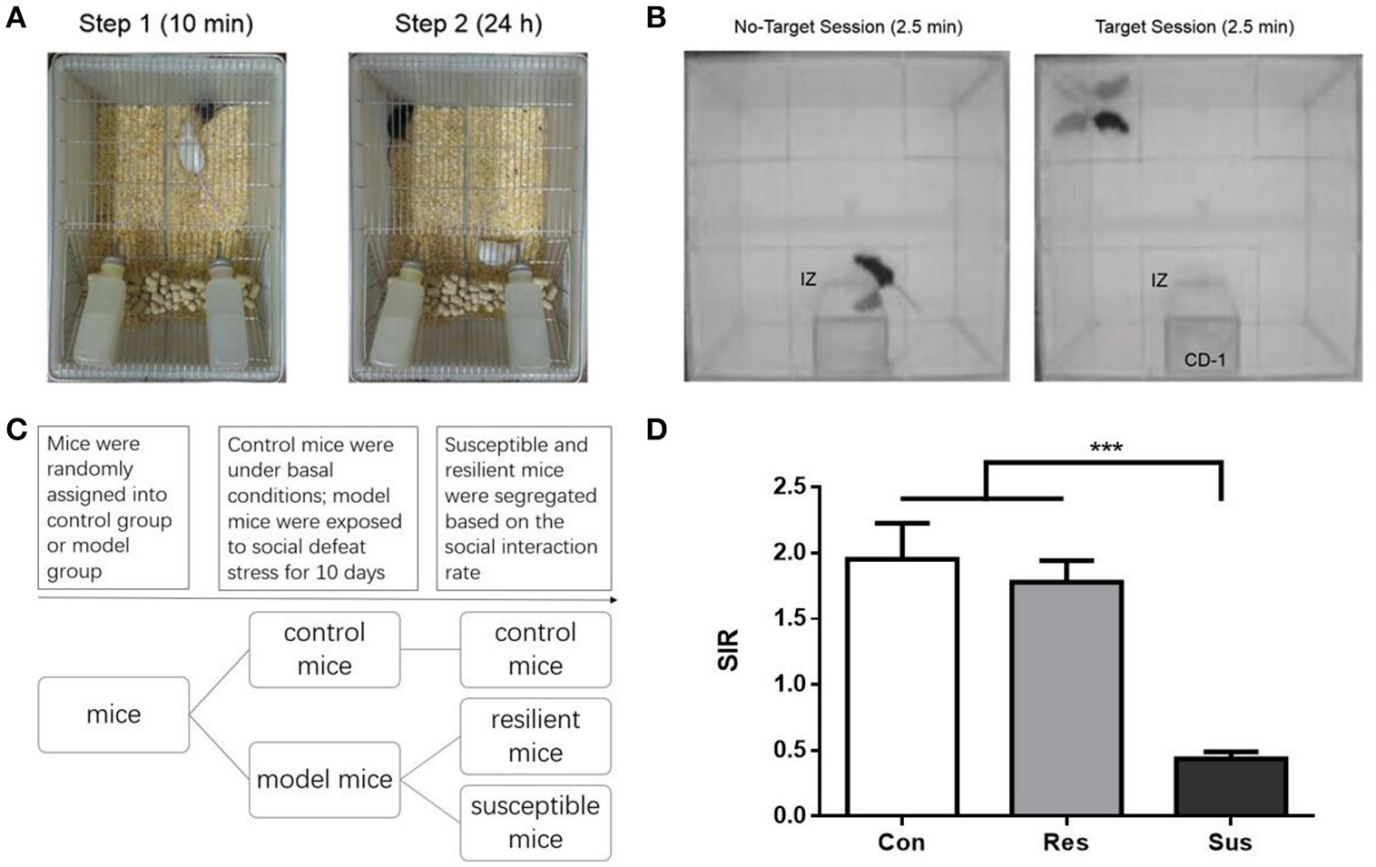
**The schematic diagram of the experimental process for chronic social defeat stress and social interaction test as well as the social interaction ratio in the social interaction test. (A)** The schematic diagram of the experimental process for chronic social defeat stress. **(B)** The schematic diagram of the experimental process for social interaction test. **(C)** The experimental procedure of the first experiment. **(D)** The social interaction rate in the social interaction test [*n*_(*Con*)_ = 20; *n*_(*Res*)_ = 18; *n*_(*Sus*)_ = 20]. All data are shown as mean ± s.e.m. ^***^P < 0.001. Con, Control; Res, Resilient; Sus, Susceptible.

The social defeat stress was conducted as previously described (Han et al., 2014). Briefly, every C57BL/6J mouse was individually introduced to the home cage of an unfamiliar aggressive CD-1 resident mouse for 5–10 min and exposed to physical defeat, after which it was housed together with the CD-1, but separated by a perforated plastic divider to allow for visual, olfactory and auditory contact for the remainder of 24 h. On the next day, the exposed mouse was transferred into a new cage that resided another unfamiliar aggressive CD-1 mouse. During the whole period of social defeat, these mice were subject to social defeat for 10 consecutive days (exposure to 10 different CD-1 mice). Control mice were kept alone in transparent plastic cages during the 10 days. For each pair of mice, the two transparent plastic cages were placed next to each other. This allowed for visual, olfactory, and auditory contact, without any physical contact. The schematic diagram of the experimental setting is shown in Figure 1A.

### Drugs

Escitalopram oxalate was obtained from Stru Chem (Suzhou, Jiangsu, P.R. China). After social defeat exposure, all animals were kept alone in home cages during the next 4 weeks with no stress. During the 4 weeks, susceptible mice were injected daily with escitalopram (10 mg/kg) or its vehicle (0.9% NaCl) intraperitoneally. Resilient mice were injected daily with the vehicle and the control mice received no injections.

### Social interaction test

Social interaction test was performed on day 11 and day 39, in a clean open arena (42 × 42 cm). Each test consisted of two 2.5 min sessions, separated by an interval of 30–60 s. In the first (also called No-Target) session, the C57BL/6J mouse was introduced to the arena with an empty mesh cage (10 × 6 cm) at one of its sides. In the second (Target) session, a mesh cage with an unfamiliar CD-1 mouse replaced the empty cage. Mesh cages allowed for visual and olfactory interactions (but no physical contact) between the test and target mice. The area of 26 × 14 cm, meaning 8 cm around the mesh cage, was defined as the interaction zone (IZ, Figure 1B). The time spent in the IZ was measured. The social interaction rate (SIR) represents the Target session time spent in the IZ divided by the No-Target session time spent in the IZ. Susceptible and resilient mice were segregated based on the SIR: mice with scores < 1 were defined as “susceptible,” and those with scores ≥1 were defined as “resilient.”

### Enzyme-linked immunosorbent assay

After social interaction test, blood collection was performed. Mice were anesthetized with ether and blood was withdrawn from retro-orbital plexus using capillary tubes. Blood was collected between 15:00 and 16:00 to avoid possible effects of circadian variations on serum corticosterone levels. Samples were collected into a separator tube and after clot formation, centrifuged at 3,000 × g for 10 min. Serum was extracted and stored at −20°C until use. The concentration of corticosterone was measured with enzyme-linked immunosorbent assay (ELISA) kits (ABCAM, ab108821) following the manufacturer's protocol. According to the sensitivity and precision of the assay, the minimum detectable dose of corticosterone was typically around 0.3 ng/mL and the intra-assay and inter-assay coefficient of variation was 5 and 7.1%, respectively. Each sample was run together with its duplicate. The mean value for each standard and sample is therefore based on two results, and the concentrations of samples were calculated from the standard curve.

### Western blotting

After decapitation, the hippocampus and hypothalamus were quickly dissected and stored at −80°C until use. The hippocampus and hypothalamus were ultrasonically disrupted in RIPA lysis buffer [50 mM Tris (pH 7.4), 150 mM NaCl, 1% Triton X-100, 1% sodium deoxycholate, 0.1% sodium dodecyl sulfonate, sodium orthovanadate, sodium fluoride, ethylene diamine tetraacetic acid, leupeptin, PMSF] followed by centrifugation at 12,000 × g for 20 min. For cytosolic and nuclear extraction, NE-PER Nuclear and Cytoplasmic Extraction Reagents (Thermo Scientific, #78833) were used following the manufacturer's protocol. The protein level was measured using the Pierce BCA Protein Assay Kit (Thermo Scientific, Rockford, IL, USA). Samples were separated on 12% acrylamide gels and then transferred onto polyvinylidene fluoride membranes. After blocking with 5% skim milk in tris-buffered-saline with tween (TBST, 20 mM Tris-HCl, pH 7.5, 150 mM NaCl, and 0.05% Tween-20) at 4°C for 2 h, the membranes were incubated with the primary antibody, anti-Glucocorticoid Receptor antibody (1:200, ABCAM, ab2768), horseradish peroxidase (HRP)-rabbit-anti-β-Actin (1:1,000, Cell Signal Technology, #12620), or anti-Histone H3 Antibody (1:1,000, Cell Signal Technology, #9715) a 4°C overnight. Then, the blots were washed in TBST and incubated in the appropriate secondary antibody, HRP-goat-anti-mouse IgG (1:10,000, Jackson ImmunoResearch Laboratories, Inc., West Grove, PA, USA) or HRP-goat-anti-rabbit IgG (1:10,000, Jackson) at 4°C for 2 h. Western blot images were captured on an ImageQuant LAS4000 Mini Image Analyzer (GE Healthcare, Buckinghamshire, UK) and the band levels were quantified using Quantity One, version 4.62.

### Quantitative real-time PCR

The hypothalamus was homogenized using Trizol. The total RNA was then reverse-transcribed using iScript™ cDNA Synthesis Kit (Bio-Rad, CA, USA) and the resulting cDNA were analyzed in quantitative polymerase chain reactions (qPCR). SYBR green detection was used according to the manufacturer's protocol (Bio-Rad, CA, USA). The 2^−ΔΔCT^ method was then used to convert ΔCT values to mRNA fold changes relative to the control group. The mRNA level of *Crf* were normalized with glyceraldehyde-3-phosphatedehydrogenase (GAPDH) mRNA level to exclude effects of varying RNA amounts. Primer sequences are as follows: Mus *Crf*, forward: GGG AGT CAT CCA GTT GTT T, reverse: GAG CTT ACA CAT TTC GTC C; Mus *Gapdh*, forward: AAA TGG TGA AGG TCG GTG TG, reverse: AGG TCA ATG AAG GGG TCG TT.

### Experimental procedure

We conducted the first experiment (Figure 1C) to investigate the individual differences in the HPA axis response and GR protein expression between susceptible and resilient mice after exposure to social defeat stress for 10 days. We conducted the second experiment (**Figure 4A**) to observe, if escitalopram could counteract behavioral abnormalities, HPA axis response and GR protein expression of susceptible mice. We conducted the third experiment (**Figure 4A**) to study the individual difference of GR protein translocation in the hippocampus between susceptibility and resilience as well as the effect of escitalopram on GR protein translocation in the hippocampus of susceptible mice.

### Statistical analysis

The data are presented as the mean ± standard error (SEM). All statistical analyses were performed and all graphs plotted using the GraphPad Prism 6.01 (GraphPad Software Inc., San Diego, CA, USA). The statistical significance of differences between groups was analyzed with a one-way analysis of variance (ANOVA) followed by the Tukey HSD. The statistical significance level was set at *p* < 0.05.

## Results

### Social interaction test after chronic social defeat stress

After exposure to social defeat stress, we carried out social interaction test to differentiate defeated mice. Only ~53% of the defeated C57 mice (*n* = 38) were classified as susceptible ones (*n* = 20) that exhibit social avoidance and the rest (*n* = 18) were identified as resilient ones that fail to develop social avoidance (Figure 1D). Besides, control mice and resilient mice behaved similarly [*F*_(2, 55)_ = 19.36, *p* = 0.7982] and both exhibited significantly higher SIR than susceptible mice [*F*_(2, 55)_ = 19.36, control mice vs. susceptible mice, *p* < 0.0001; resilient mice vs. susceptible mice, *p* < 0.0001, Figure 1D]. In our study susceptible mice showed obvious social avoidance, while the behavior of resilient mice resembled this of normal control mice.

### Individual differences in the HPA axis response after chronic social defeat stress

**Figure 2:**
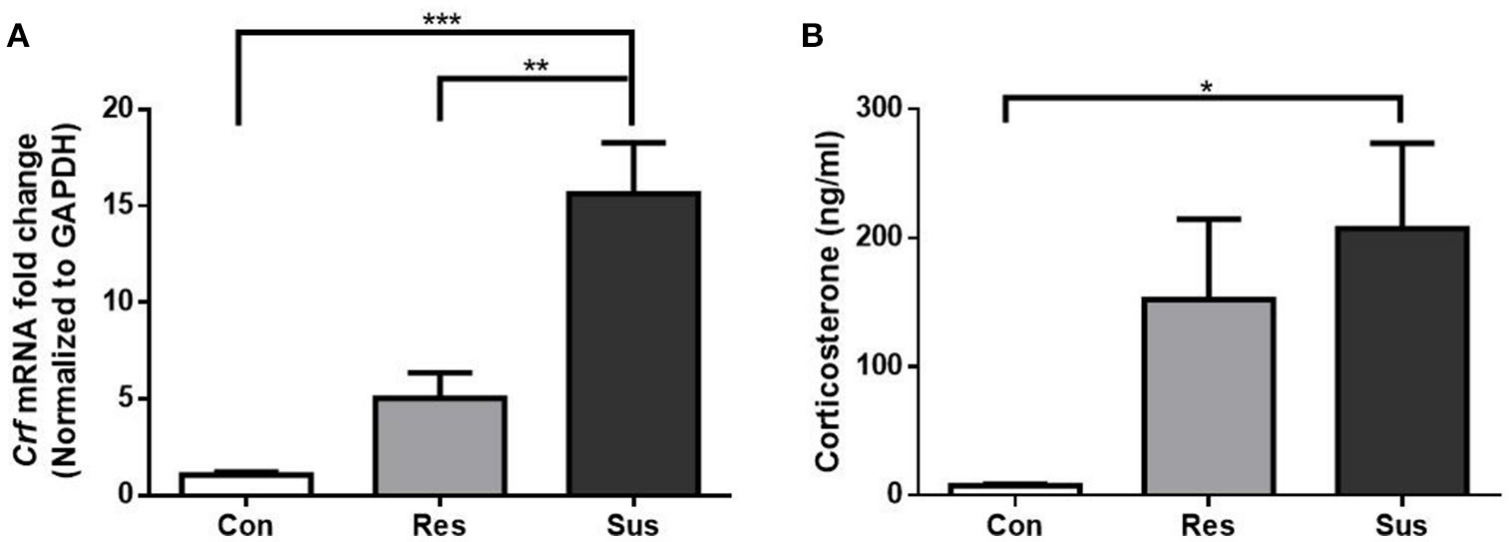
**Chronic social defeat stress induced hypercortisolemia in susceptible mice. (A)** mRNA expression of *Crf* in the hypothalamus of mice after exposure to social defeat stress (*n* = 6/group). **(B)** Serum CORT concentration of mice after exposure to social defeat stress (*n* = 7/group). All data are shown as mean ± s.e.m. ^*^P < 0.05, ^**^P < 0.01, ^***^P < 0.001. Con, Control; Res, Resilient; Sus, Susceptible.

To monitor the HPA axis response, we detected the mRNA expression of *Crf* in the hypothalamus and serum CORT concentration of mice. After exposure to social defeat stress, susceptible mice showed significantly more *Crf* mRNA expression than control and resilient mice [*F*_(2, 15)_ = 19.63, control mice vs. susceptible mice, *p* < 0.0001; resilient mice vs. susceptible mice, *p* = 0.0014; control mice vs. resilient mice, *p* = 0.2539, Figure 2A], and significantly more serum CORT than control mice [*F*_(2, 18)_ = 3.855, control mice vs. susceptible mice, *p* = 0.0380; resilient mice vs. susceptible mice, *p* = 0.7411; control mice vs. resilient mice, *p* = 0.1551, Figure 2B], which indicated that social defeat stress induced hypercortisolemia in susceptible mice. Despite some gains in *Crf* mRNA expression and serum CORT concentration in resilient mice, the differences were not significant as compared to control mice. The data demonstrate that HPA axis of resilient mice was activated slightly less than this of susceptible mice.

### Individual differences in GR protein expression after chronic social defeat stress

**Figure 3:**
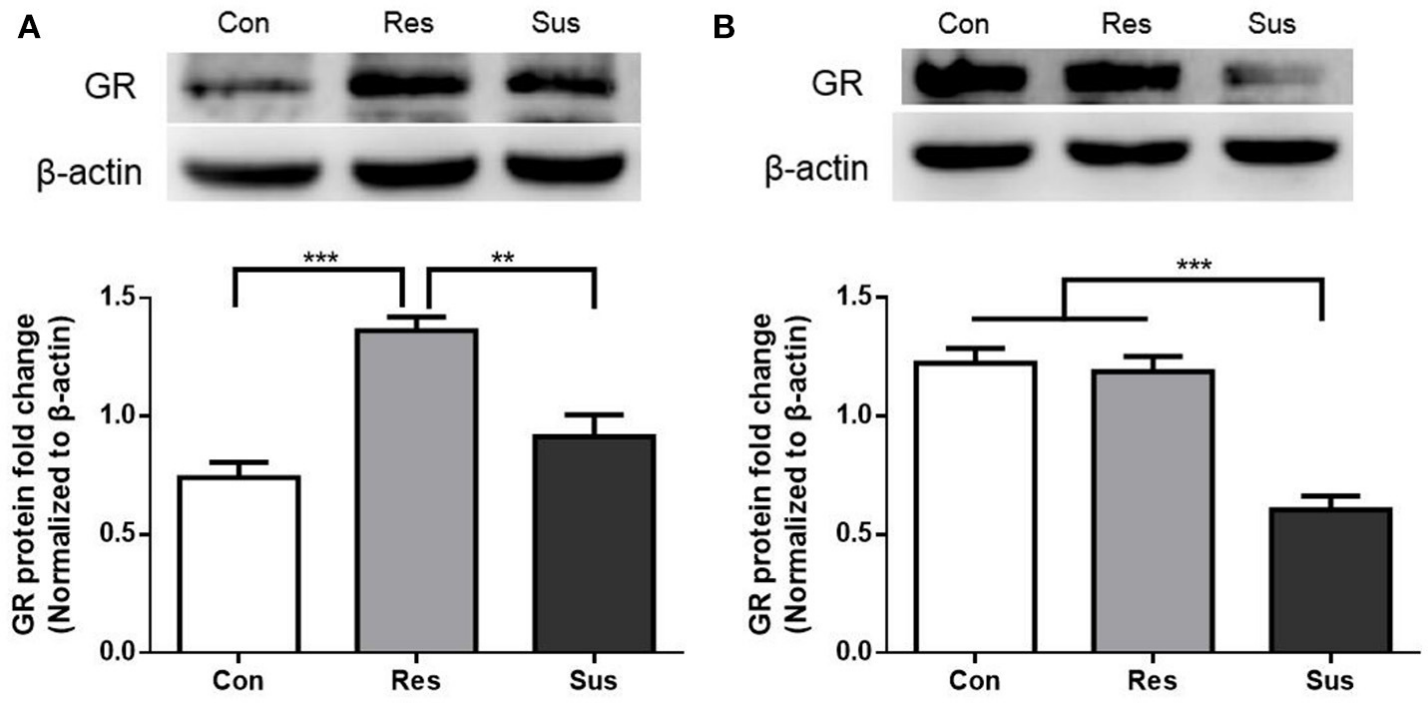
**GR protein expression were differently regulated in resilient and susceptible mice. (A)** GR protein expression in the hippocampus of mice after exposure to social defeat stress (*n* = 7/group). **(B)** GR protein expression in the hypothalamus of mice after exposure to social defeat stress (*n* = 7/group). All data are shown as mean ± s.e.m. ^**^P < 0.01, ^***^P < 0.001. Con, Control; Res, Resilient; Sus, Susceptible.

After exposure to social defeat stress, resilient mice showed significantly more GR protein expression than control and susceptible mice in the hippocampus [*F*_(2, 18)_ = 19.18, control mice vs. susceptible mice, *p* = 0.2382; resilient mice vs. susceptible mice, *p* = 0.0011; control mice vs. resilient mice, *p* < 0.0001, Figure 3A]. This result indicates that in response to social defeat stress, there was sufficient GR protein expression in resilient mice. Moreover, susceptible mice showed significantly less GR protein expression than control and resilient mice in the hypothalamus [*F*_(2, 18)_ = 32.23, control mice vs. susceptible mice, *p* < 0.0001; resilient mice vs. susceptible mice, *p* < 0.0001; control mice vs. resilient mice, *p* = 0.9119, Figure 3B]. These results indicate that susceptible mice exhibited significantly less GR protein expression than resilient mice in the hippocampus and hypothalamus.

### Social interaction test after 4 weeks of treatment

**Figure 4:**
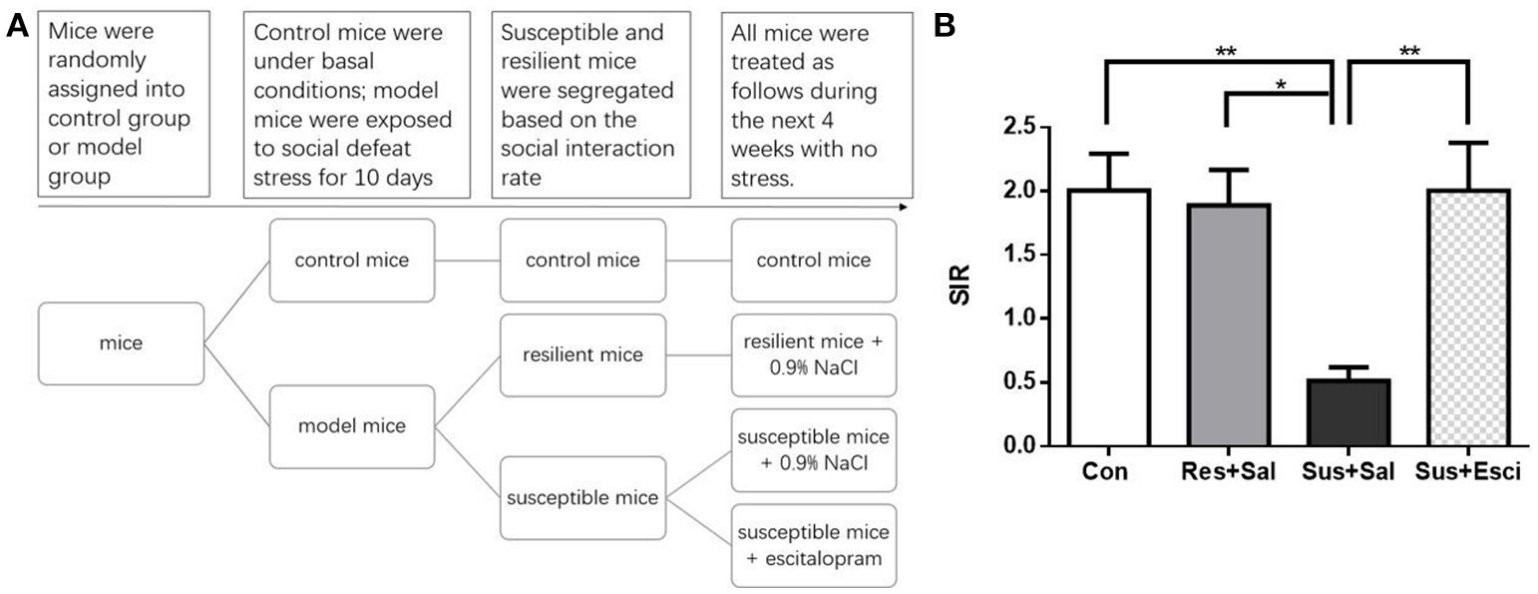
**Escitalopram could counteract the social avoidance behavior. (A)** The experimental procedure of the second and third experiment. **(B)** The social interaction rate in the social interaction test after 4 weeks treatment [*n*_(*Con*)_ = 20; *n*_(*Res*+*Sal*)_ = 12; *n*_(*Sus*+*Sal*)_ = 13; *n*_(*Sus*+*Esci*)_ = 16]. All data are shown as mean ± s.e.m. ^*^P < 0.05, ^**^P < 0.01. Con, Control; Res+Sal, Resilient+Saline; Sus+Sal, Susceptible+Saline; Sus+Esci, Susceptible+Escitalopram.

After 4 weeks of treatment, escitalopram alleviated the social avoidance behavior of susceptible mice. However, susceptible mice administered with vehicle still exhibited significantly lower SIR than other groups [*F*_(3, 57)_ = 5.431, susceptible+saline mice vs. control mice, *p* = 0.0036; susceptible+saline mice vs. resilient+saline mice, *p* = 0.0226; susceptible+saline mice vs. susceptible+escitalopram mice, *p* = 0.0059, Figure 4B]. The data indicate that social avoidance behavior can continue for at least 4 weeks and that escitalopram could counteract the abnormal behavior induced by chronic social defeat stress.

### The HPA axis response after 4 weeks of treatment

**Figure 5:**
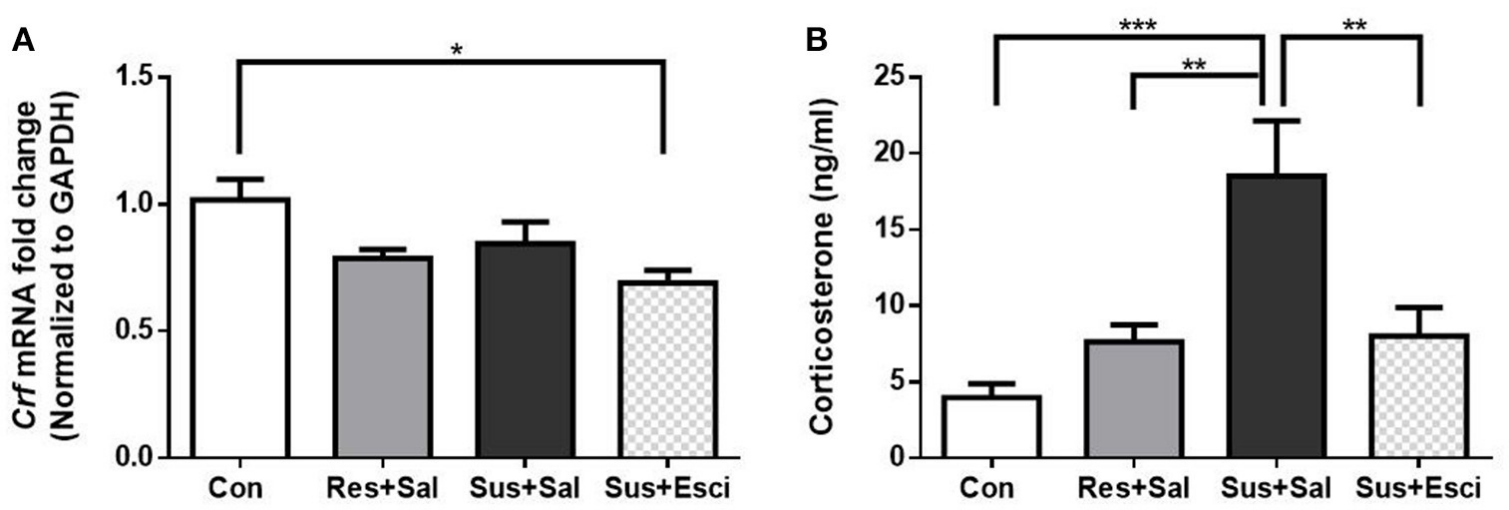
***Crf* mRNA expression in hypothalamus and serum CORT after 4 weeks treatment. (A)** mRNA expression of *Crf* in the hypothalamus of mice after 4 weeks treatment [*n*_(*Con*)_ = 6; *n*_(*Res*+*Sal*)_ = 5; *n*_(*Sus*+*Sal*)_ = 6; *n*_(*Sus*+*Esci*)_ = 6]. **(B)** Serum CORT concentration of mice after 4 weeks treatment [*n*_(*Con*)_ = 7; *n*_(*Res*+*Sal*)_ = 5; *n*_(*Sus*+*Sal*)_ = 4; *n*_(*Sus*+*Esci*)_ = 6]. All data are shown as mean ± s.e.m. ^*^P < 0.05, ^**^P < 0.01, ^***^P < 0.001. Con, Control; Res+Sal, Resilient+Saline; Sus+Sal, Susceptible+Saline; Sus+Esci, Susceptible+Escitalopram.

The *Crf* mRNA expression in the hypothalamus of susceptible mice subsided 4 weeks after the last social defeat stress [*F*_(3, 19)_ = 4.191, susceptible+saline mice vs. control mice, *p* = 0.3006, Figure 5A]. Besides, it is noteworthy that susceptible mice administered with escitalopram exhibited significantly lower *Crf* mRNA expression than control mice in the hypothalamus [*F*_(3, 19)_ = 4.191, susceptible+escitalopram mice vs. control mice, *p* = 0.0129, Figure 5A]. The data indicate that escitalopram can attenuate the *Crf* mRNA expression in the hypothalamus. Additionally, even though serum CORT of the model groups also decreased to some extent, the serum CORT of susceptible mice administered with vehicle still kept a high level, compared with other groups [*F*_(3, 18)_ = 10.15, susceptible+saline mice vs. control mice, *p* = 0.0002; susceptible+saline mice vs. resilient+saline mice, *p* = 0.0063; susceptible+saline mice vs. susceptible+escitalopram mice, *p* = 0.0062, Figure 5B].

### GR protein expression after 4 weeks of treatment

**Figure 6:**
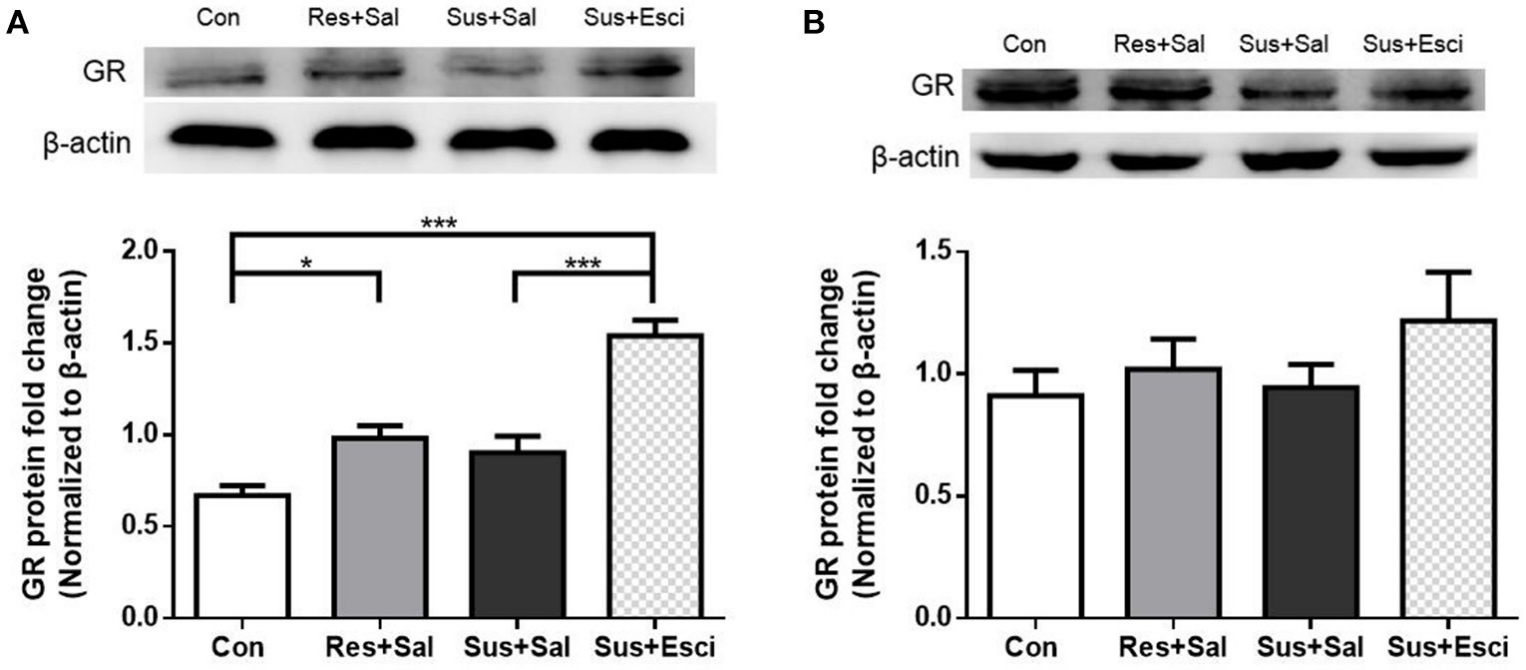
**Antidepressant could enhance the GR protein expression in hippocampus in susceptible mice. (A)** GR protein expression in the hippocampus of mice after 4 weeks treatment (*n* = 6/group). **(B)** GR protein expression in the hypothalamus of mice after 4 weeks treatment (*n* = 6/group). All data are shown as mean ± s.e.m. ^*^P < 0.05, ^***^P < 0.001. Con, Control; Res+Sal, Resilient+Saline; Sus+Sal, Susceptible+Saline; Sus+Esci, Susceptible+Escitalopram.

Susceptible mice administered with escitalopram expressed significantly more GR protein in the hippocampus than those administered with vehicle [*F*_(3, 20)_ = 23.73, susceptible+escitalopram mice vs. susceptible+saline mice, *p* < 0.0001], and showed significantly more GR protein expression than control mice to face past distressing events, just like resilient mice [*F*_(3, 20)_ = 23.73, susceptible+escitalopram mice vs. control mice, *p* < 0.0001; resilient+saline mice vs. control mice, *p* = 0.0398, Figure 6A], which indicates that the antidepressant could enhance the GR protein expression in the hippocampus. Combined with the result in Figure 4, this indicates that in response to a bitter memory from the past, such as social defeat, there was sufficient GR protein expression in resilient mice and susceptible ones administered with antidepressant, all of which engaged in active social interaction. However, there were no significant differences of GR protein expression in the hypothalamus [*F*_(3, 20)_ = 1.002, Figure 6B]. Thus, we next examined the GR protein translocation in the hippocampus by detecting its levels in the cytosolic and nuclear compartments.

### Cytosolic and nuclear protein expression of GR in the hippocampus after chronic social defeat stress

**Figure 7:**
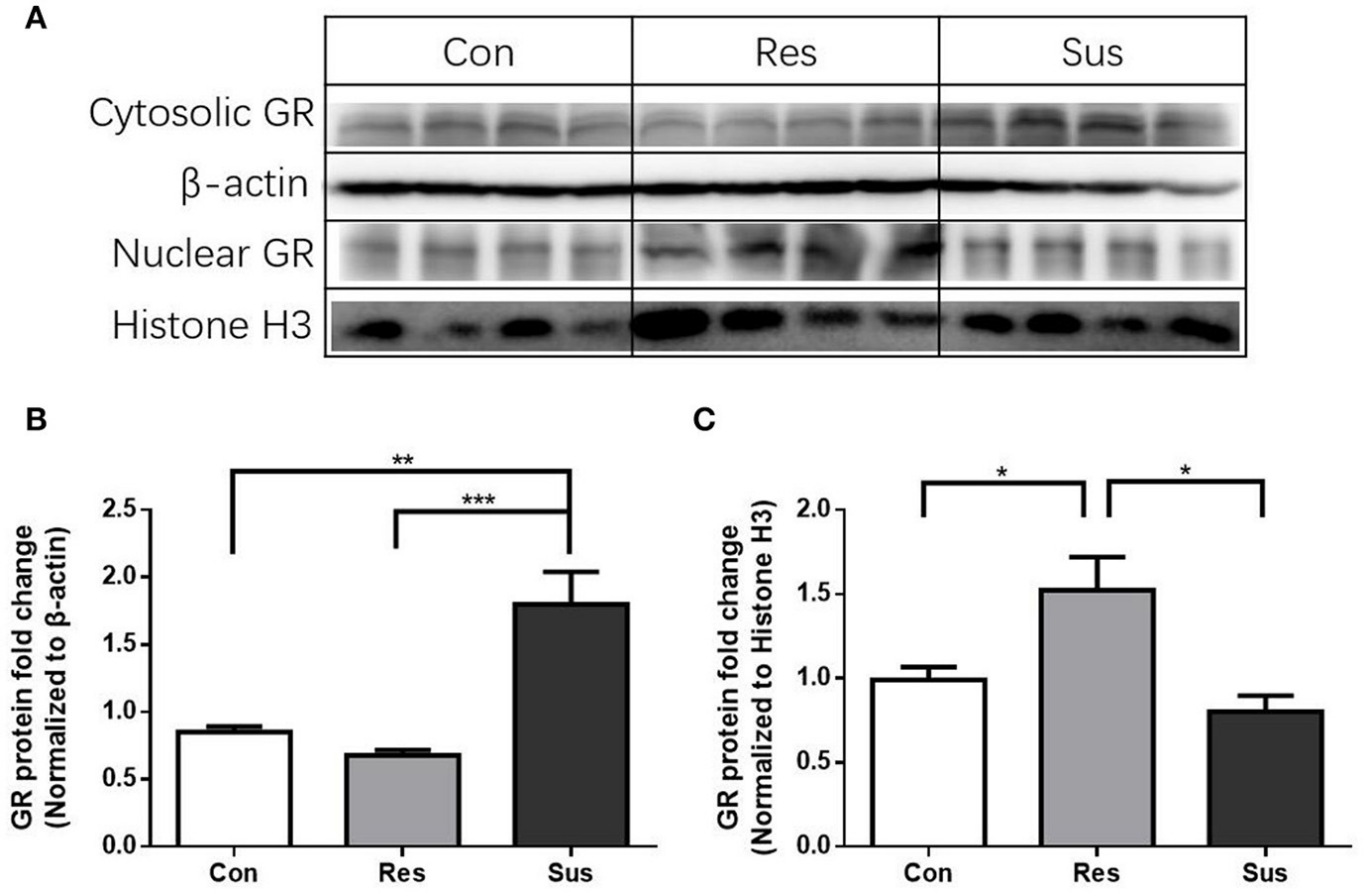
**More nuclear translocation of GR in the hippocampus was found in resilient mice than susceptible mice after chronic social defeat stress. (A)** Representative western blots showing cytosolic and nuclear protein expression of GR. **(B)** Quantification of cytosolic protein expression of GR in the hippocampus after chronic social defeat stress (*n* = 4/group). **(C)** Quantification of nuclear protein expression of GR in the hippocampus after chronic social defeat stress (*n* = 4/group). All data are shown as mean ± s.e.m. ^*^P < 0.05, ^**^P < 0.01, ^***^P < 0.001. Con, Control; Res, Resilient; Sus, Susceptible.

After exposure to social defeat stress, susceptible mice exhibited significantly more cytosolic GR protein expression than control and resilient mice [*F*_(2, 9)_ = 17.90, control mice vs. susceptible mice, *p* = 0.0029; resilient mice vs. susceptible mice, *p* = 0.0009; control mice vs. resilient mice, *p* = 0.6774, Figures 7A,B], while resilient mice showed significantly more nuclear GR protein expression than control and susceptible mice in the hippocampus [*F*_(2, 9)_ = 7.821, control mice vs. susceptible mice, *p* = 0.6000; resilient mice vs. susceptible mice, *p* = 0.0104; control mice vs. resilient mice, *p* = 0.0479, Figures 7A,C]. These results indicate that there was more nuclear translocation of GR in the hippocampus of resilient mice than in that of susceptible mice.

### Cytosolic and nuclear protein expression of GR in the hippocampus after 4 weeks of treatment

**Figure 8:**
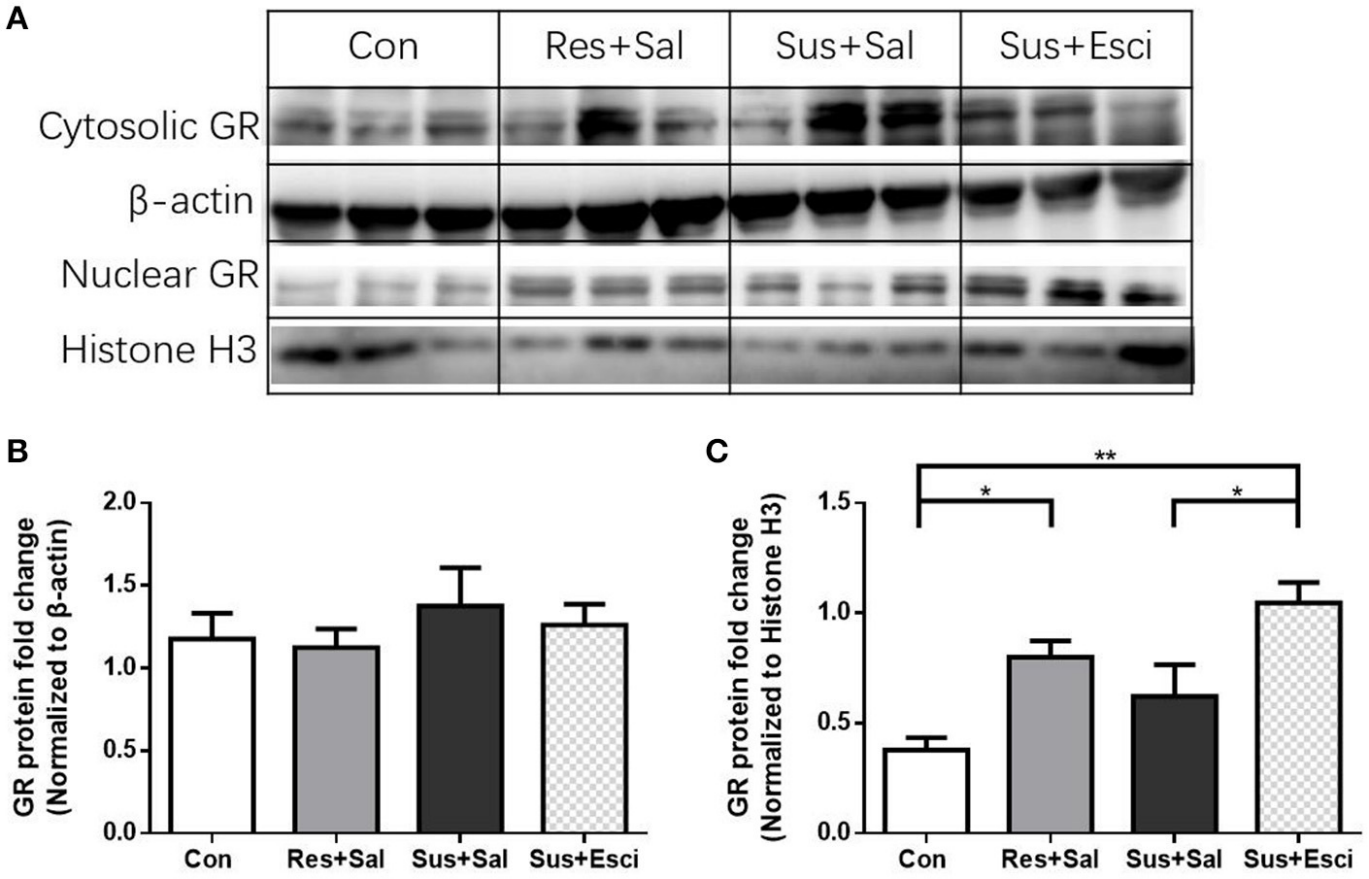
**Escitalopram could increase nuclear translocation of GR in hippocampus of susceptible mice. (A)** Representative western blots showing cytosolic and nuclear protein expression of GR. **(B)** Quantification of cytosolic protein expression of GR in the hippocampus after 4 weeks treatment (*n* = 4/group). **(C)** Quantification of nuclear protein expression of GR in the hippocampus after 4 weeks treatment (*n* = 4/group). All data are shown as mean ± s.e.m. ^*^P < 0.05, ^**^P < 0.01. Con, Control; Res+Sal, Resilient+Saline; Sus+Sal, Susceptible+Saline; Sus+Esci, Susceptible+Escitalopram.

There were no significant differences of cytosolic GR protein expression in the hippocampus 4 weeks after the last social defeat stress [*F*_(3, 12)_ = 0.4462, Figures 8A,B]. However, susceptible mice administered with escitalopram expressed significantly more nuclear GR protein in the hippocampus than those administered with vehicle [*F*_(3, 12)_ = 8.432, susceptible+escitalopram mice vs. susceptible+saline mice, *p* = 0.0410] and, similar to resilient mice, showed significantly more nuclear GR protein expression than control mice, [*F*_(3, 12)_ = 8.432, susceptible+escitalopram mice vs. control mice, *p* = 0.0019; resilient+saline mice vs. control mice, *p* = 0.0419, Figures 8A,C]. The data imply that escitalopram could increase nuclear translocation of GR and ultimately reverse social withdrawal behaviors.

## Discussion

It is worth noting that resilient individuals have a stronger survival instinct and a more active attitude toward coping with extreme stress. As described above, an advantage of chronic social defeat stress is that it can be utilized to study “resilience,” a subset of mice fail to develop behavioral or metabolic disturbances in contrast to susceptible mice. In our study, susceptible mice showed obvious social avoidance which could last at least 4 weeks, while resilient mice displayed normal behavior, similar to unstressed control mice.

As has been reported, coping style in response to stress is associated with how the neuroendocrine systems are activated (Zozulya et al., 2008). Any behavioral abnormalities may be associated with the HPA axis response. In our study, after exposure to social defeat stress for 10 days, susceptible mice showed significantly more *Crf* mRNA expression than control and resilient mice, and significantly more serum CORT than control mice. And even though the *Crf* mRNA expression of susceptible mice subsided 4 weeks after the last social defeat stress, the serum CORT in this group still remained at a high level. Increased basal corticosterone levels following chronic stress exposure have been reported in many studies (Sapolsky, 1992; Bartolomucci et al., 2005; Keeney et al., 2006; Schmidt et al., 2007). Similarly to our study, Wood et al. found that rats with reduced CORT release developed proactive resisting behaviors (Wood et al., 2010) after exposure to social defeat stress. However, in contrast to us, they found a decrease in *Crf* mRNA in susceptible rats, but not in resilient rats, as compared with controls, and so they speculated that the decrease in *Crf* mRNA may be due to increased translation of *Crf* mRNA into protein in susceptible rats. Consistent with our results, another recent work (Elliott et al., 2010) revealed a significant increase in *Crf* mRNA levels in susceptible, but not in resilient mice. They found that the *Crf* gene was hyper-methylated, which prevented *Crf* mRNA synthesis in the resilient mice, while DNA methylation levels were decreased at four specific CpGs in the *Crf* promoter in susceptible mice (Elliott et al., 2010). In the study of Krishnan et al. (2007), daytime levels of serum CORT between control, susceptible and resilient mice were similar on day 11, the possible reason for which may be that the intensity of the social defeat stress of their experiment was much lower than that of our study. However, on day 39, they found that daytime levels of serum corticosterone in resilient mice were significantly higher than in susceptible mice, although neither of them was significantly different from controls, which was very interesting and puzzled us.

In response to stress, glucocorticoids, as a consequence of HPA axis activation, interact with GR which is expressed at high levels in hippocampus and hypothalamus. GR function as transcription factors to mediate negative feedback of HPA axis (Ulrich-Lai and Herman, 2009) to maintain the neuroendocrine systems in balance. In our study, susceptible mice exhibited significantly less GR protein expression than resilient mice in the hippocampus and hypothalamus after exposure to social defeat stress for 10 consecutive days and the alteration in the hippocampus could last for at least 4 weeks. Additional evidence has reported that resilient responses to stress are associated with decreased DNA methylation of the *Gr* gene, higher levels of GR expression, and greater feedback inhibition of the HPA axis (Weaver et al., 2005), which is consistent with our study, even though low-maternal-care stress early in life was utilized to induce different phenotype in adults, which is different from our study. Aside from that, changes in GR nuclear translocation may also play a vital role in HPA axis hyperactivity in depression (Schmidt et al., 2009; Anacker et al., 2011). In our study, increased nuclear translocation of GR in the hippocampus in resilient mice contributed to reduced activation of the HPA axis and active social interaction. One study (Hartmann et al., 2012b) showed that a certain kind of transgenic mice displayed a more active stress-coping behavior, as well as lower adrenal weights and basal corticosterone levels. These results also hinted to a less vulnerable phenotype and an enhanced negative glucocorticoid feedback within the HPA axis of those transgenic mice, possibly modulated by increased GR sensitivity. Another study (Hartmann et al., 2012a) by the same group revealed that another kind of transgenic mice exhibited high stress vulnerability and enhanced neuroendocrine response to stress, which was possibly mediated by reduced GR sensitivity. Furthermore, we discovered a decreased translocation of GR that could mediate the vulnerable phenotype of susceptible mice in the present study. After all, our susceptible mice appear to match this phenotype. In addition, the GR which is distributed in the limbic system, is also involved in the neural process of emotional responses (Fitzsimons et al., 2013). The down-regulation of GR expression and translocation in hippocampus may not only reflect the maladaptive responses of HPA axis, but is also a likely aspect of the neurobiological component of individual differences in emotionality to stress.

Further, we found that escitalopram could reverse social withdrawal behaviors in susceptible mice. Actually, at the beginning of our experimental study resilient mice were also administered with escitalopram. The data showed no difference in social interaction test between resilient mice administered with vehicle and those administered with escitalopram [*F*_(4, 66)_ = 4.348, resilient+saline mice vs. resilient+escitalopram mice, *p* > 0.9999, Supplementary Figure 1]. Thus, in our follow-up experiment, in which we focused on the mechanism responsible for the effect of escitalopram on susceptible mice, we omitted the group of resilient mice treated with escitalopram. Furthermore, we found that escitalopram could decrease the serum CORT, increase GR protein expression and nuclear translocation in the hippocampus in susceptible mice. These results indicate that the attenuation of serum CORT, the up-regulation of GR and the enhancement of GR nuclear translocation in the hippocampus play an important role in resilience to chronic social defeat stress. That is to say, the differential GR protein expression and translocation in the hippocampus could very well be what determines between susceptibility vs. resilience to chronic social defeat stress. In conclusion, resilient and susceptible mice use different coping strategies in the face of stress, which ultimately result in different consequences we can observe.

## Author contributions

All authors mentioned in the paper have significantly contributed to the research. QH and LY established the chronic social defeat stress-induced mice model of depression and performed some behavioral tests, enzyme-linked immunosorbent assay, western blotting and quantitative real-time PCR. HH, YW, RY, and JW performed some behavioral tests. QL and JY designed the research and write the paper. GW and AP revised the paper.

### Conflict of interest statement

The authors declare that the research was conducted in the absence of any commercial or financial relationships that could be construed as a potential conflict of interest. The reviewer TK and handling Editor declared their shared affiliation, and the handling Editor states that the process nevertheless met the standards of a fair and objective review.

## References

[B1] Anacker C. Zunszain P. A. Carvalho L. A. Pariante C. M. (2011). The glucocorticoid receptor: pivot of depression and of antidepressant treatment? Psychoneuroendocrinology 36, 415–425. 10.1016/j.psyneuen.2010.03.007 20399565 PMC3513407

[B2] Bartolomucci A. Palanza P. Sacerdote P. Panerai A. E. Sgoifo A. Dantzer R.. (2005). Social factors and individual vulnerability to chronic stress exposure. Neurosci. Biobehav. Rev. 29, 67–81. 10.1016/j.neubiorev.2004.06.009 15652256

[B3] Becker C. Zeau B. Rivat C. Blugeot A. Hamon M. Benoliel J. J. (2008). Repeated social defeat-induced depression-like behavioral and biological alterations in rats: involvement of cholecystokinin. Mol. Psychiatry 13, 1079–1092. 10.1038/sj.mp.4002097 17893702

[B4] Berton O. McClung C. A. Dileone R. J. Krishnan V. Renthal W. Russo S. J. (2006). Essential role of BDNF in the mesolimbic dopamine pathway in social defeat stress. Science 311, 864–868. 10.1126/science.1120972 16469931

[B5] Charney D. S. (2004). Psychobiological mechanisms of resilience and vulnerability: implications for successful adaptation to extreme stress. Am. J. Psychiatry 161, 195–216. 10.1176/appi.ajp.161.2.195 14754765

[B6] Compton W. M. Conway K. P. Stinson F. S. Grant B. F. (2006). Changes in the prevalence of major depression and comorbid substance use disorders in the United States between 1991-1992 and 2001-2002. Am. J. Psychiatry 163, 2141–2147. 10.1176/ajp.2006.163.12.2141 17151166

[B7] Del Giudice M. Ellis B. J. Shirtcliff E. A. (2011). The Adaptive Calibration Model of stress responsivity. Neurosci. Biobehav. Rev. 35, 1562–1592. 10.1016/j.neubiorev.2010.11.007 21145350 PMC3068241

[B8] Elliott E. Ezra-Nevo G. Regev L. Neufeld-Cohen, A. Chen A. (2010). Resilience to social stress coincides with functional DNA methylation of the Crf gene in adult mice. Nat. Neurosci. 13, 1351–1353. 10.1038/nn.2642 20890295

[B9] Feder A. Nestler E. J. Charney D. S. (2009). Psychobiology and molecular genetics of resilience. Nat. Rev. Neurosci. 10, 446–457. 10.1038/nrn2649 19455174 PMC2833107

[B10] Fitzsimons C. P. van Hooijdonk L. W. Schouten M. Zalachoras I. Brinks V. Zheng T.. (2013). Knockdown of the glucocorticoid receptor alters functional integration of newborn neurons in the adult hippocampus and impairs fear-motivated behavior. Mol. Psychiatry 18, 993–1005. 10.1038/mp.2012.123 22925833

[B11] Fuchs E. Flugge G. (2002). Social stress in tree shrews: effects on physiology, brain function, and behavior of subordinate individuals. Pharmacol. Biochem. Behav. 73, 247–258. 10.1016/S0091-3057(02)00795-5 12076743

[B12] Franklin T. B. Saab B. J. Mansuy I. M. (2012). Neural mechanisms of stress resilience and vulnerability. Neuron 75, 747–761. 10.1016/j.neuron.2012.08.016 22958817

[B13] Golden S. A. Covington H. E. Berton O. Russo S. J. (2011). A standardized protocol for repeated social defeat stress in mice. Nat. Protoc. 6, 1183–1191. 10.1038/nprot.2011.361 21799487 PMC3220278

[B14] Guidotti G. Calabrese F. Anacker C. Racagni G. Pariante C. M. Riva M. A. (2013). Glucocorticoid receptor and FKBP5 expression is altered following exposure to chronic stress: modulation by antidepressant treatment. Neuropsychopharmacology 38, 616–627. 10.1038/npp.2012.225 23169346 PMC3572458

[B15] Han Q. Yang L. Liu Y. Lv N. Yu J. Wu G.. (2014). Resiliency to social defeat stress relates to the inter-strain social interaction and is influenced by season variation. Neurosci. Lett. 561, 13–17. 10.1016/j.neulet.2013.12.045 24374287

[B16] Hartmann J. Wagner K. V. Dedic N. Marinescu D. Scharf S. H. Wang X. D.. (2012a). Fkbp52 heterozygosity alters behavioral, endocrine and neurogenetic parameters under basal and chronic stress conditions in mice. Psychoneuroendocrinology 37, 2009–2021. 10.1016/j.psyneuen.2012.04.017 22641006

[B17] Hartmann J. Wagner K. V. Liebl C. Scharf S. H. Wang X. D. Wolf M. (2012b). The involvement of FK506-binding protein 51 (FKBP5) in the behavioral and neuroendocrine effects of chronic social defeat stress. Neuropharmacology 62, 332–339. 10.1016/j.neuropharm.2011.07.041 21839098

[B18] Keeney A. Jessop D. S. Harbuz M. S. Marsden C. A. Hogg S. Blackburn-Munro R. E. (2006). Differential effects of acute and chronic social defeat stress on hypothalamic-pituitary-adrenal axis function and hippocampal serotonin release in mice. J. Neuroendocrinol. 18, 330–338. 10.1111/j.1365-2826.2006.01422.x 16629831

[B19] Krishnan V. Han M. H. Graham D. L. Berton O. Renthal W. Russo S. J.. (2007). Molecular adaptations underlying susceptibility and resistance to social defeat in brain reward regions. Cell 131, 391–404. 10.1016/j.cell.2007.09.018 17956738

[B20] Kudryavtseva N. N. Bakshtanovskaya I. V. Koryakina L. A. (1991). Social model of depression in mice of C57BL/6J strain. Pharmacol. Biochem. Behav. 38, 315–320. 10.1016/0091-3057(91)90284-9 2057501

[B21] McEwen B. S. Gould E. A. Sakai R. R. (1992). The vulnerability of the hippocampus to protective and destructive effects of glucocorticoids in relation to stress. Br. J. Psychiatry Suppl. 160, 18–23. 1389022

[B22] Mizoguchi K. Ishige A. Aburada M. Tabira T. (2003). Chronic stress attenuates glucocorticoid negative feedback: involvement of the prefrontal cortex and hippocampus. Neuroscience 119, 887–897. 10.1016/s0306-4522(03)00105-2 12809708

[B23] Nestler E. J. Hyman S. E. (2010). Animal models of neuropsychiatric disorders. Nat. Neurosci. 13, 1161–1169. 10.1038/nn.2647 20877280 PMC3750731

[B24] Pich E. M. Heinrichs S. C. Rivier C. Miczek K. A. Fisher D. A. Koob G. F. (1993). Blockade of pituitary-adrenal axis activation induced by peripheral immunoneutralization of corticotropin-releasing factor does not affect the behavioral response to social defeat stress in rats. Psychoneuroendocrinology 18, 495–507. 10.1016/0306-4530(93)90043-K 8265737

[B25] Raone A. Cassanelli A. Scheggi S. Rauggi R. Danielli B. De Montis M. G. (2007). Hypothalamus-pituitary-adrenal modifications consequent to chronic stress exposure in an experimental model of depression in rats. Neuroscience 146, 1734–1742. 10.1016/j.neuroscience.2007.03.027 17481824

[B26] Razzoli M. Carboni L. Arban R. (2009). Alterations of behavioral and endocrinological reactivity induced by 3 brief social defeats in rats: relevance to human psychopathology. Psychoneuroendocrinology 34, 1405–1416. 10.1016/j.psyneuen.2009.04.018 19482436

[B27] Russo S. J. Murrough J. W. Han M. H. Charney D. S. Nestler E. J. (2012). Neurobiology of resilience. Nat. Neurosci. 15, 1475–1484. 10.1038/nn.3234 23064380 PMC3580862

[B28] Rygula R. Abumaria N. Domenici E. Hiemke C. Fuchs E. (2006a). Effects of fluoxetine on behavioral deficits evoked by chronic social stress in rats. Behav. Brain Res. 174, 188–192. 10.1016/j.bbr.2006.07.017 16949682

[B29] Rygula R. Abumaria N. Flugge G. Fuchs E. Ruther E. Havemann-Reinecke U. (2005). Anhedonia and motivational deficits in rats: impact of chronic social stress. Behav. Brain Res. 162, 127–134. 10.1016/j.bbr.2005.03.009 15922073

[B30] Rygula R. Abumaria N. Flugge G. Hiemke C. Fuchs E. Ruther E.. (2006b). Citalopram counteracts depressive-like symptoms evoked by chronic social stress in rats. Behav. Pharmacol. 17, 19–29. 10.1097/01.fbp.0000186631.53851.71 16377960

[B31] Sapolsky R. M. (1992). Cortisol concentrations and the social significance of rank instability among wild baboons. Psychoneuroendocrinology 17, 701–709. 10.1016/0306-4530(92)90029-7 1287688

[B32] Schatzberg A. F. (2015). Anna-Monika Award Lecture, DGPPN Kongress, 2013: the role of the hypothalamic-pituitary-adrenal (HPA) axis in the pathogenesis of psychotic major depression. World J. Biol. Psychiatry 16, 2–11. 10.3109/15622975.2014.916414 24933348

[B33] Schmidt M. V. Sterlemann V. Ganea K. Liebl C. Alam S. Harbich D.. (2007). Persistent neuroendocrine and behavioral effects of a novel, etiologically relevant mouse paradigm for chronic social stress during adolescence. Psychoneuroendocrinology 32, 417–429. 10.1016/j.psyneuen.2007.02.011 17449187

[B34] Schmidt M. V. Sterlemann V. Wagner K. Niederleitner B. Ganea K. Liebl C. (2009). Postnatal glucocorticoid excess due to pituitary glucocorticoid receptor deficiency: differential short- and long-term consequences. Endocrinology 150, 2709–2716. 10.1210/en.2008-1211 19213843

[B35] Schuhmacher A. Lennertz L. Wagner M. Hofels S. Pfeiffer U. Guttenthaler V. (2013). A variant of the neuronal amino acid transporter SLC6A15 is associated with ACTH and cortisol responses and cognitive performance in unipolar depression. Int. J. Neuropsychopharmacol. 16, 83–90. 10.1017/S1461145712000223 22475622

[B36] Silva M. T. Galvao T. F. Martins S. S. Pereira M. G. (2014). Prevalence of depression morbidity among Brazilian adults: a systematic review and meta-analysis. Rev. Bras. Psiquiatr. 36, 262–270. 10.1590/1516-4446-2013-1294 25119639

[B37] Ulrich-Lai Y. M. Herman J. P. (2009). Neural regulation of endocrine and autonomic stress responses. Nat. Rev. Neurosci. 10, 397–409. 10.1038/nrn2647 19469025 PMC4240627

[B38] Weaver I. C. Champagne F. A. Brown S. E. Dymov S. Sharma S. Meaney M. J.. (2005). Reversal of maternal programming of stress responses in adult offspring through methyl supplementation: altering epigenetic marking later in life. J. Neurosci. 25, 11045–11054. 10.1523/JNEUROSCI.3652-05.2005 16306417 PMC6725868

[B39] Wood S. K. Walker H. E. Valentino R. J. Bhatnagar S. (2010). Individual differences in reactivity to social stress predict susceptibility and resilience to a depressive phenotype: role of corticotropin-releasing factor. Endocrinology 151, 1795–1805. 10.1210/en.2009-1026 20160137 PMC2850230

[B40] Yu T. Guo M. Garza J. Rendon S. Sun X. L. Zhang W.. (2011). Cognitive and neural correlates of depression-like behaviour in socially defeated mice: an animal model of depression with cognitive dysfunction. Int. J. Neuropsychopharmacol. 14, 303–317. 10.1017/S1461145710000945 20735879 PMC3432579

[B41] Zozulya A. A. Gabaeva M. V. Sokolov O. Y. Surkina I. D. Kost N. V. (2008). Personality, coping style, and constitutional neuroimmunology. J. Immunotoxicol. 5, 221–225. 10.1080/15476910802131444 18569393

