# Supplementary material for: Differential GR Expression and Translocation in the Hippocampus Mediates Susceptibility vs. Resilience to Chronic Social Defeat Stress
Source: Front Neurosci. 2017 May 23;11:287. doi: 10.3389/fnins.2017.00287 (PMC5440566; doi:10.3389/fnins.2017.00287)
Supplement: Supplementary file 1 [file Image1.PDF]

Supplementary Material

Differential GR expression and translocation in the hippocampus mediates susceptibility versus resilience to chronic social defeat stress

Qiu-Qin Han[#a], Liu Yang[#a], Hui-Jie Huang[#a], Ya-Lin Wang[a], Rui Yu[a], Jing Wang[a], Adam Pilot[a], Gen-Cheng Wu[a], Qiong Liu*[b, c], Jin Yu*[a]

*** Correspondence:** Qiong Liu: liuqiong@fudan.edu.cn; Jin Yu: yujin@shmu.edu.cn

Supplementary Figure

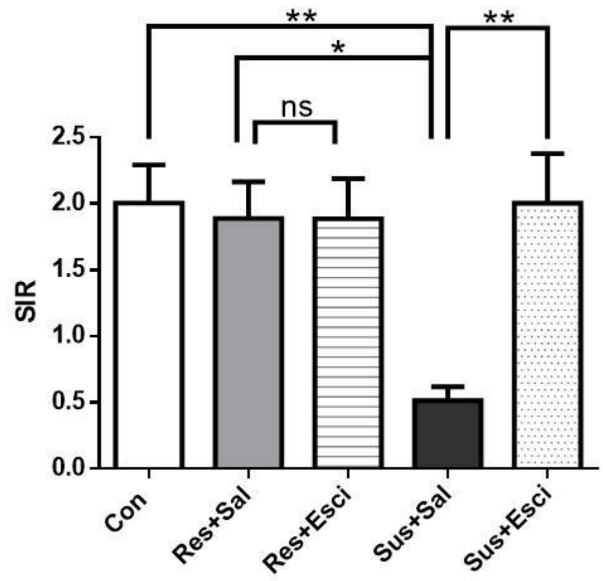

Supplementary Figure 1. Escitalopram alleviated the social avoidance behavior in susceptible mice and did not influence the social interaction in resilient mice ($n_{(Con)}=20$; $n_{(Res+Sal)}=12$; $n_{(Res+Esci)}=10$; $n_{(Sus+Sal)}=13$; $n_{(Sus+Esci)}=16$). All data are shown as mean ± s.e.m. *P < 0.05, **P < 0.01. Con: Control; Res+Sal: Resilient+Saline; Res+Esci: Resilient+Escitalopram; Sus+Sal: Susceptible+Saline; Sus+Esci: Susceptible+Escitalopram.
